# Surfactant protein-D predicts prognosis of interstitial lung disease induced by anticancer agents in advanced lung cancer: a case control study

**DOI:** 10.1186/s12885-017-3285-6

**Published:** 2017-05-02

**Authors:** Kota Nakamura, Motoyasu Kato, Takehito Shukuya, Keita Mori, Yasuhito Sekimoto, Hiroaki Ihara, Ryota Kanemaru, Ryo Ko, Rina Shibayama, Ken Tajima, Ryo Koyama, Naoko Shimada, Osamu Nagashima, Fumiyuki Takahashi, Shinichi Sasaki, Kazuhisa Takahashi

**Affiliations:** 1 Department of Respiratory Medicine, Juntendo University Graduate School of Medicine, 3-1-3, Hongo, Bunkyo-ku, Tokyo, 113-8431 Japan; 2 Department of Respiratory Medicine, Juntendo University Urayasu Hospital, 2-1-1, Tomioka, Urayasu, Chiba 273-0021 Japan; 3 Clinical Trial Coordination Office, Shizuoka Cancer Center, 1007 Shimonagakubo, Nagaizumi-cho, Suntou-gun, Shizuoka 411-8777 Japan

**Keywords:** Interstitial lung disease, Drug-induced interstitial lung disease, Lung cancer

## Abstract

**Background:**

Interstitial lung diseases induced by anticancer agents (ILD-AA) are rare adverse effects of anticancer therapy. However, prognostic biomarkers for ILD-AA have not been identified in patients with advanced lung cancer. Our aim was to analyze the association between serum biomarkers sialylated carbohydrate antigen Krebs von den Lungen-6 (KL-6) and surfactant protein D (SP-D), and clinical characteristics in patients diagnosed with ILD-AA.

**Methods:**

Between April 2011 and March 2016, 1224 advanced lung cancer patients received cytotoxic agents and epidermal growth factor receptor tyrosine kinase inhibitors at Juntendo University Hospital and Juntendo University Urayasu Hospital. Of these patients, those diagnosed with ILD-AA were enrolled in this case control study. ΔKL-6 and ΔSP-D were defined as the difference between the levels at the onset of ILD-AA and their respective levels prior to development of ILD-AA. We evaluated KL-6 and SP-D at the onset of ILD-AA, ΔKL-6 and ΔSP-D, the risk factors for death related to ILD-AA, the chest high resolution computed tomography (HRCT) findings, and survival time in patients diagnosed with ILD-AA.

**Results:**

Thirty-six patients diagnosed with ILD-AA were enrolled in this study. Among them, 14 patients died of ILD-AA. ΔSP-D in the patients who died was significantly higher than that in the patients who survived. However, ΔKL-6 did not differ significantly between the two groups. Moreover, ΔSP-D in patients who exhibited diffuse alveolar damage was significantly higher than that in the other patterns on HRCT. Receiver operating characteristic curve analysis was used to set the optimal cut off value for ΔSP-D at 398 ng/mL. Survival time for patients with high ΔSP-D (≥ 398 ng/mL) was significantly shorter than that for patients with low ΔSP-D. Multivariate analysis revealed that ΔSP-D was a significant prognostic factor of ILD-AA.

**Conclusions:**

This is the first research to evaluate high ΔSP-D (≥ 398 ng/mL) in patients with ILD-AA and to determine the risk factors for ILD-AA in advanced lung cancer patients. ΔSP-D might be a serum prognostic biomarker of ILD-AA. Clinicians should evaluate serum SP-D during chemotherapy and should carefully monitor the clinical course in patients with high ΔSP-D.

## Background

Drug-induced interstitial lung disease (D-ILD) is one of the most common adverse events caused by anticancer agents. Patients with advanced lung cancer typically receive chemotherapy. If patients develop interstitial lung disease (ILD) induced by anticancer agents (ILD-AA), clinicians cannot continue using the same anticancer agents to treat these patients. Thus, the development of ILD-AA can be critical for a patient’s prognosis. A pre-existing interstitial shadow on chest high resolution computed tomography (HRCT) and past smoking history are known as significant risk factors [1–3]. The incidence of ILD-AA has been reported to be 1% to 5% for several anticancer agents [4–6]. Moreover, the incidence of ILD-AA has been reported to be more than 20% in patients with a usual interstitial pneumonia pattern identified on HRCT [1, 6].

Many serum markers, including sialylated carbohydrate antigen Krebs von den Lungen-6 (KL-6), surfactant protein D (SP-D), and surfactant protein A (SP-A), are often used for evaluation of D-ILD. KL-6 is typically elevated in patients with idiopathic interstitial pneumonias (IIPs), hypersensitivity pneumonia (HP), and connective tissue diseases associated with interstitial pneumonia (CTD-IP). Moreover, elevated serum KL-6 is useful in classification of D-ILD patterns on HRCT [7]. In this study, serum KL-6 in patients with diffuse alveolar damage (DAD) and chronic interstitial pneumonia (CIP) was significantly higher than that in those with other patterns of D-ILD. However, KL-6 is also a tumor marker and a useful indicator of the progression of lung, breast, and pancreatic cancer [8]. Therefore, it is difficult to determine whether elevated KL-6 levels are caused by the development of D-ILD or by cancer progression. Surfactant proteins are produced by type II alveolar epithelial cells. SP-D is elevated in patients with IIPs, CTD-IP, radiation pneumonia, and D-ILD and is considered a useful serum marker in any type of ILD [9, 10].

We focused on the difference in SP-D levels before and after the onset of ILD-AA. Few studies have investigated the association between SP-D and D-ILD compared with other serum markers, particularly KL-6. We aimed to investigate the relationships among serum biomarkers, including KL-6 and SP-D, the patterns of ILD-AA as assessed using HRCT scans, and the prognosis of patients with advanced lung cancer.

## Methods

### Study population

**Fig. 1:**
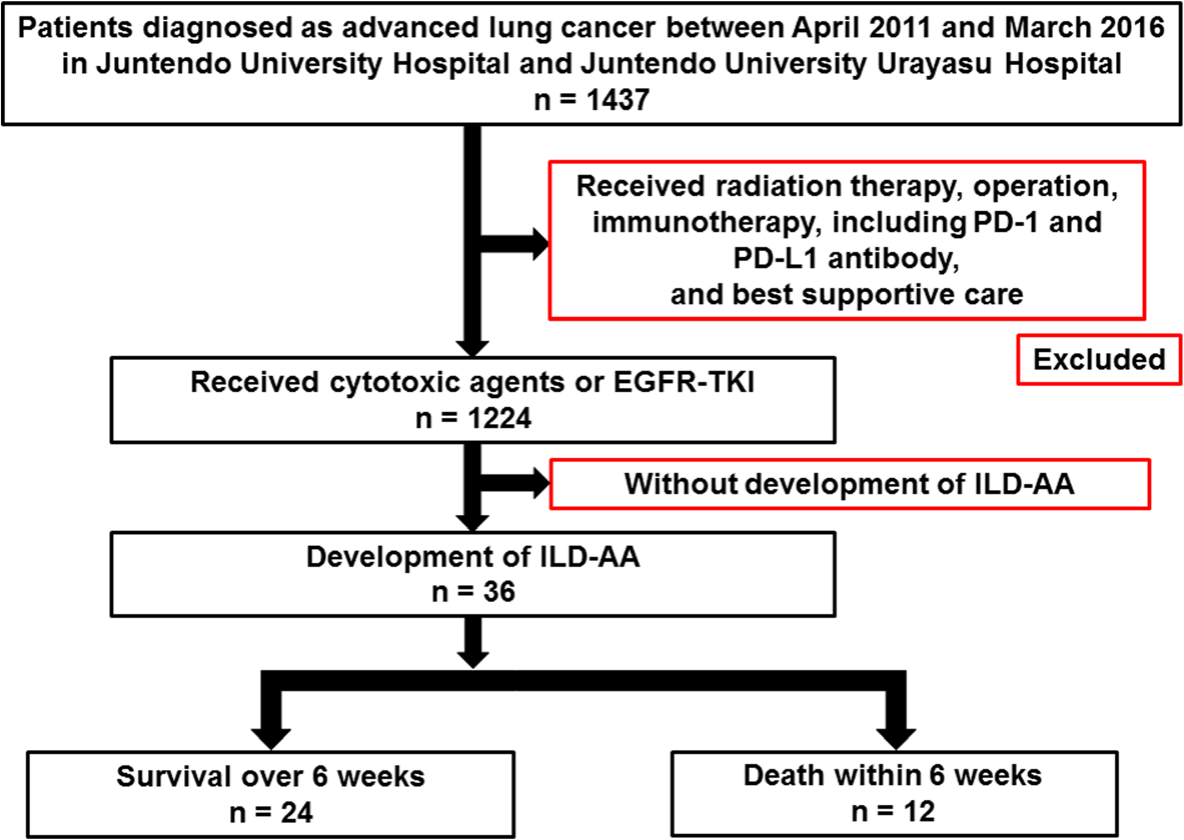
Study patients

Between April 2011 and March 2016, 1437 patients were diagnosed with advanced lung cancer at Juntendo University Hospital and Juntendo University Urayasu Hospital, and 1224 of them received chemotherapy, including cytotoxic agents and epidermal growth factor receptor (EGFR) tyrosine kinase inhibitors. Thirty-six of these patients diagnosed with ILD-AA (13 at Juntendo University Hospital and 23 at Juntendo University Urayasu Hospital) were enrolled in this case control study. All patients were diagnosed with ILD-AA on the basis of HRCT findings and elevated levels of serum markers, including KL-6 and SP-D. At the time of diagnosis with ILD-AA, there was no evidence of infection, heart failure, or renal failure. Patients receiving immunotherapy, operation, and thoracic radiotherapy were excluded from the study. Patients who died within 6 weeks of the onset of ILD-AA were included in the death group and those who survived over 6 weeks were included in the survival group (Fig. 1). The study protocol was approved by the Juntendo University Ethical Committee and registered under number 16-051. Owing to the retrospective nature of the research, the Ethical Committee waived the requirement for informed consent.

### Serum biomarkers

The serum biomarkers KL-6 and SP-D were measured in all of the patients prior to the diagnosis of ILD-AA and at the time of ILD-AA diagnosis. Serum KL-6 and SP-D were measured using a sandwich enzyme-linked immunosorbent assay, with KL-6 and SP-D antibodies (SRL, Inc. Tokyo, Japan). The cut off values for serum KL-6 and SP-D were set at 500 U/mL and 110 ng/mL, respectively. The differences (ΔKL-6 and ΔSP-D) were calculated as the value of KL-6 and SP-D levels at the onset of ILD-AA minus the respective values prior to ILD-AA.

### Chest HRCT findings

At the onset of ILD-AA, all of the patients underwent HRCT, which was performed with 2-mm collimation at 10-mm intervals, from the lung apex to the lung base. Based on the HRCT results at the time of diagnosis with ILD-AA, patients were classified into four groups: HP, DAD, CIP, and organized pneumonia/eosinophilic pneumonia (OP/EP). HP was defined as only ground glass opacity (GGO) without traction bronchiectasis and honeycombing. DAD was characterized by extensive bilateral GGO or consolidation with traction bronchiectasis. CIP was defined as evidence of fibrosis, including subpleural reticular shadow, honeycombing, or reticular shadows on the bronchovascular bundles. OP/EP was characterized by peribronchial and/or subpleural consolidation of the bronchovascular bundles.

### Evaluation

We evaluated KL-6 and SP-D at the onset of ILD-AA, ΔKL-6 and ΔSP-D, the risk factors for survival and death, the HRCT findings, and the effect of high or low ΔKL-6 and ΔSP-D on survival.

### Statistical analysis

We used the chi-square test, Fisher’s exact test, and the Wilcoxon two-sample test to compare patient characteristics and the frequency of ILD-AA. Receiver operating characteristic (ROC) curve analysis was used to determine the cut off levels for ΔKL-6 and ΔSP-D. The sensitivity, specificity, and diagnostic accuracy of the cut off levels were evaluated. KL-6 and SP-D levels were compared with HRCT classifications by using the Kruskal-Wallis test. Differences in survival time were analyzed using a log-rank test. The Cox proportional hazards model was used to calculate the hazard ratio (HR). Logistic regression analysis was used to estimate the risk of death due to ILD-AA. Univariate and multivariate analyses were performed to identify risk factors associated with death due to ILD-AA. A *p* value of less than 0.05 was considered significant. All statistical analyses were performed using SPSS version 19.0 for Windows (Chicago, IL, USA).

## Results

### Patient’s characteristics

Thirty-six patients diagnosed with ILD-AA during treatment with anticancer agents were enrolled in this study. Patient’s characteristics are shown in Table 1. All of the patients were Japanese. Patient’s median age was 71 years (range: 53-87 years). Nine (25%) patients were women, 31 (86.1%) were smokers, 27 (75%) had good performance status (PS = 0, 1), 21 (58.3%) had pre-existing interstitial shadow on HRCT, and 23 (63.9%) had emphysema on HRCT. Twenty-four patients had adenocarcinoma, nine had small cell carcinoma, and three had squamous cell carcinoma. Five patients had sensitive EGFR mutation. No patients had the EML4/ALK fusion gene. All 36 patients received several types of chemotherapy regimens. Suspected regimens are shown as Table 2. Patients without the EGFR mutation were treated with cytotoxic chemotherapy, including pemetrexed plus platinum agents (*n* = 7), pemetrexed monotherapy (*n* = 3), carboplatin plus paclitaxel (*n* = 4), albumin combined with paclitaxel (*n* = 1), docetaxel (*n* = 5), bevacizumab with carboplatin plus paclitaxel (*n* = 2), etoposide plus platinum agents (*n* = 4), amurubicin (*n* = 3), and nogitecan (*n* = 2). There was no association between death related to ILD-AA and any specific anticancer agent. Moreover, there was no evidence of cancer progression or carcinomatous lymphangitis by HRCT findings and tumor marker elevation in all patients. Fourteen patients died of respiratory failure related to ILD-AA within 6 weeks.

**Table 1:** Patient’s characteristics

	*n* = 36
Age	
≥ 71	17
≤ 70	19
Sex	
Men	27
Women	9
Smoking history	
Yes	31
No	5
Performance status	
0–1	27
2–4	9
Histological type	
Adenocarcinoma	24
Squamous cell	3
Small cell	9
Disease stage	
IIIB	5
IV	26
Post-operative recurrence	5
EGFR mutation	
Yes	5
No	31
With pre-existing interstitial shadow	
Yes	21
No	15
With emphysema	
Yes	23
No	13

*Abbreviation: EGFR* epidermal growth factor receptor

**Table 2:** Chemotherapy regimens

Regimen	*n* = 36
SCLC	*n* = 9
CDDP/CBDCA + VP-16	4
NGT	2
AMR	3
NSCLC (EGFR mutation wild type)	*n* = 22
CBDCA + PAC + BEV	2
CBDCA + PAC	4
CBDCA + nab-PAC	1
CDDP/CBDCA + PEM	7
PEM	3
DOC	5
NSCLC (EGFR mutation sensitive)	*n* = 5
gefitinib	2
erlotinib	3

*Abbreviations: EGFR* epidermal growth factor receptor, *SCLC* small cell lung cancer, *NSCLC* non-small cell lung cancer, *CDDP* cisplatin, *CBDCA* carboplatin, *VP-16* etoposide, *NGT* nogitecan, *AMR* amrubicin, *PAC* paclitaxel, *BEV* bevacizumab, *nab-PAC* albumin-binding paclitaxel, *PEM* pemetrexed, *DOC* docetaxel

### Association between serum markers and ILD-AA

**Fig. 2:**
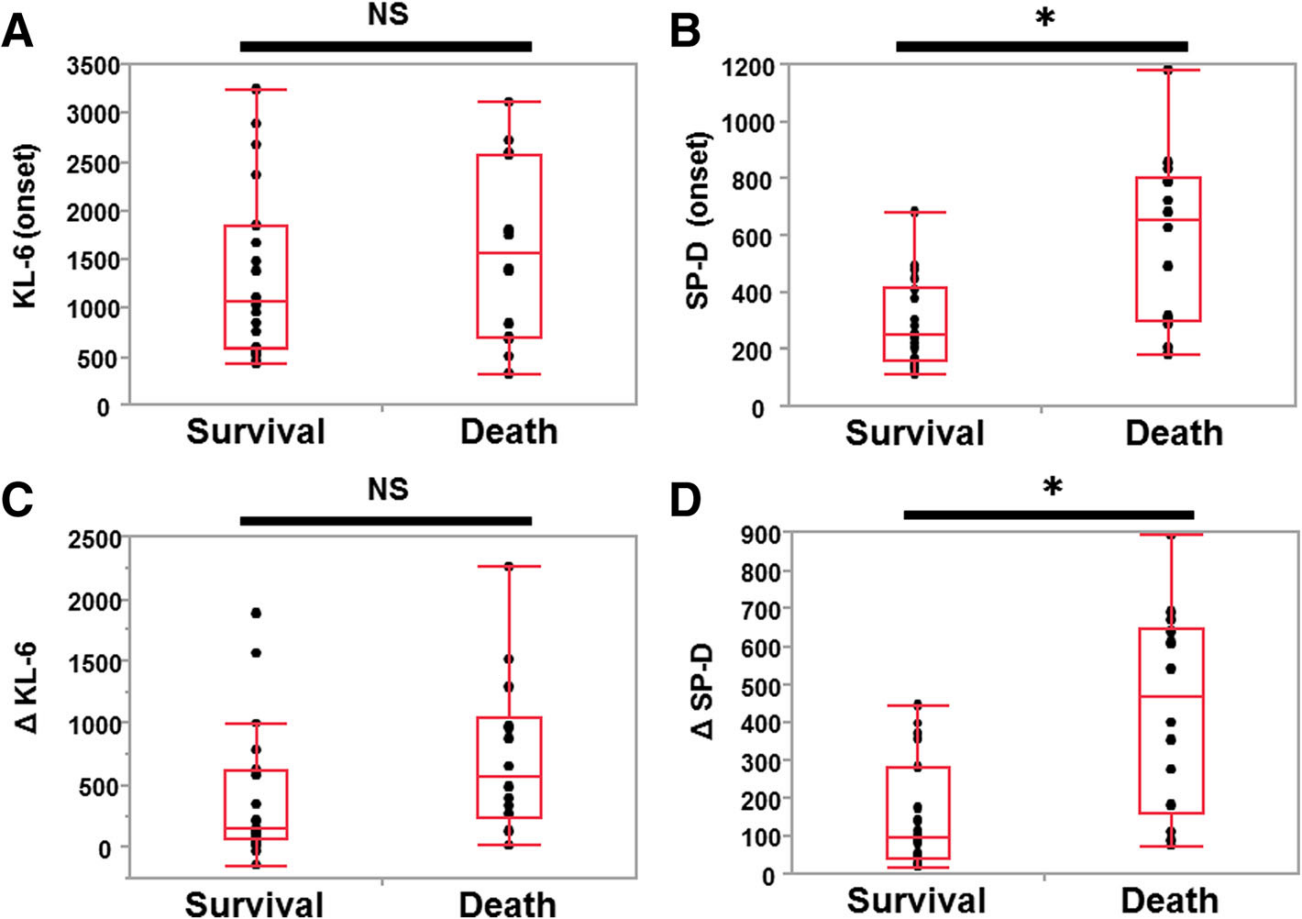
Association between survival and serum markers. Difference in serum markers between the survival and the death group; **a** Krebs von den Lungen-6 (KL-6) and **b** surfactant protein-D (SP-D) at the onset of interstitial lung disease induced by anticancer agents (ILD-AA), and **c** ΔKL-6, and **d** ΔSP-D. The Box-whisker plots demonstrate the 25th and 75th percentages, the median (horizontal line within the box), and the 10th and 90th percentages (whiskers). * *p* < 0.01 by Mann-Whitney U- test. NS: no significant difference

Serum KL-6 and SP-D levels were analyzed prior to and at the onset of ILD-AA in all 36 patients. The median serum KL-6 and SP-D values prior to ILD-AA were 1100 U/mL (range: 327–3328 U/mL) and 314 ng/mL (range: 22–393 ng/mL), respectively. When compared to the values prior to ILD-AA, serum KL-6 levels in 31 patients (86.1%) and serum SP-D in all patients (100%) at the onset of ILD-AA were increased from prior to ILD-AA. Figure 2 shows the serum KL-6 and SP-D levels at the onset of ILD-AA by outcome. When patients in the survival group were compared to those in the death group, the serum SP-D levels in the death group at the onset of ILD-AA were significantly higher than those in the survival group (Mann–Whitney U-test; *p* = 0.002, Fig. 2b). However, serum KL-6 levels did not differ significantly between the two groups (*p* = 0.833, Fig. 2a). When ΔSP-D was compared between the two groups, ΔSP-D in the death group was significantly higher than that in the survival group (*p* = 0.0008, Fig. 2d); however, ΔKL-6 did not differ considerably between the two groups (*p* = 0.282, Fig. 2c).

### Association between serum markers and HRCT findings

**Fig. 3:**
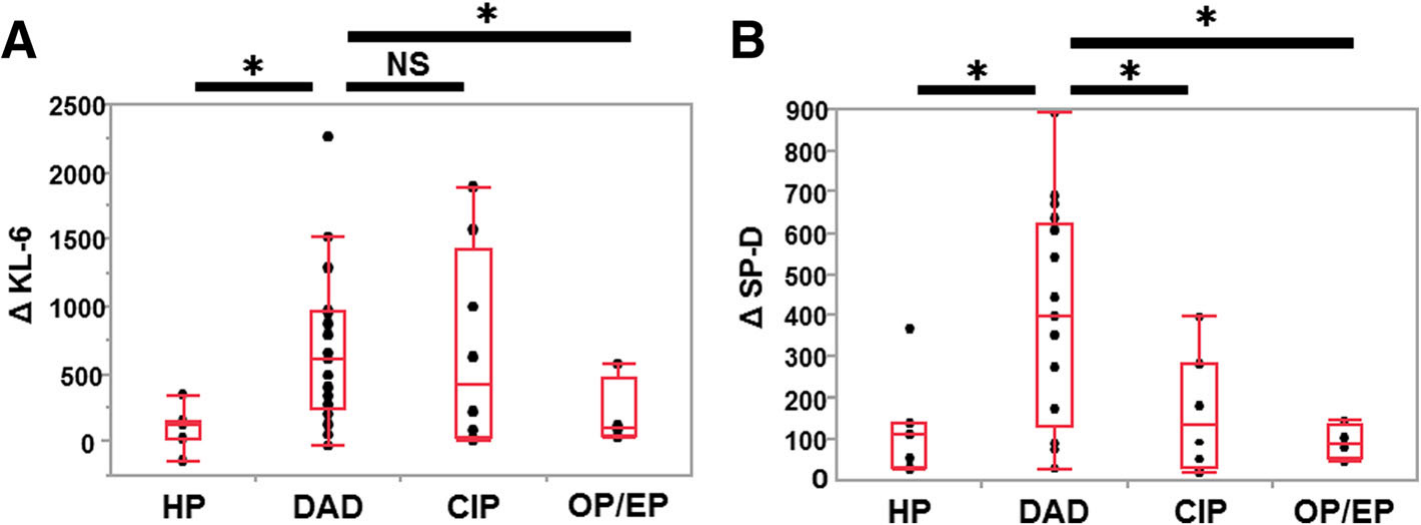
Association between chest high resolution computed tomography pattern and Δ serum markers. Different chest high resolution computed tomography (HRCT) patterns, including diffuse alveolar damage (DAD), chronic interstitial pneumonia (CIP), organized pneumonia/eosinophilic pneumonia (OP/EP), and hypersensitivity pneumonia (HP), and **a** ΔKL-6 and **b** ΔSP-D. The Box-whisker plots show the 25th and 75th percentiles, the median (horizontal line within the box), and the 10th and 90th percentiles (whiskers). * *p* < 0.01 by Mann-Whitney U- test. NS: no significant difference

We also evaluated the association between HRCT patterns at the onset of ILD-AA and serum biomarkers. In the study population, HRCT patterns were as follows: HP (*n* = 6), DAD (*n* = 14), CIP (*n* = 7), and OP/EP (*n* = 9). Figure 3a and b show ΔKL-6 and ΔSP-D according to ILD-AA patterns. As shown in Fig. 3a, ΔKL-6 in patients with the DAD pattern was significantly higher than in patients with the HP or OP/EP patterns (DAD-HP*, p* = 0.019; DAD-OP/EP*, p* = 0.033); however, there was no significant difference in ΔKL-6 between the DAD and CIP (*p* = 0.962). In patients with DAD, ΔSP-D was significantly elevated when compared to the three other types of ILD-AA (DAD-HP, *p* = 0.011; DAD-CIP, *p* = 0.022; DAD-OP/EP, *p* = 0.029; Fig. 3b); however, there were no significant differences in ΔSP-D among the three other types of ILD-AA. Therefore, ΔSP-D was significantly related to the DAD pattern. Although 21 patients had pre-existing interstitial shadow on HRCT, there was no significant difference between HRCT patterns and pre-existing interstitial shadow. Moreover, pre-existing interstitial shadow in all patients were categorized into IIPs. Of these patients, 14 patients with pre-existing interstitial shadow were clinically diagnosed with idiopathic pulmonary fibrosis (IPF). The subtypes of IIPs in pre-existing interstitial shadow were not associated with ILD-AA patterns. Then, of the 14 patients with IPF, the serum SP-D levels in 5 patients and serum KL-6 levels in 12 patients were elevated before the diagnosis of ILD-AA. However, there was no significant association between change in serum markers and pre-existing IPF (data not shown). Moreover, there were no findings of carcinomatous lymphangitis, including the thickening of the bronchovascular bundle, interlobular septa, and centrilobular micro nodules on HRCT in any patients.

### Association between serum markers change and clinical course

**Fig. 4:**
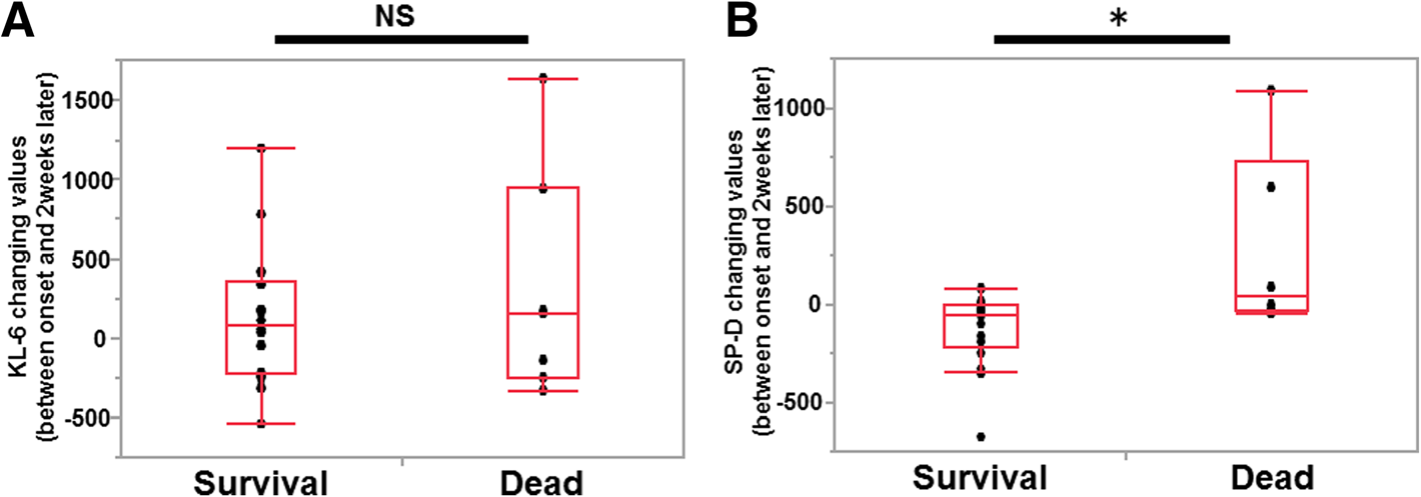
Association between survival and change of serum markers (between the onset of ILD-AA and 2 weeks after diagnosis with ILD-AA). **a** Difference between KL-6 change and survival. **b** Difference between SP-D change and survival. The Box-whisker plots show the 25th and 75th percentiles, the median (horizontal line within the box) and the 10th and 90th percentiles (whiskers). * *p* < 0.01 by Mann-Whitney U- test. NS: no significant difference

Serum KL-6 and SP-D levels were also measured 2 weeks after the onset of ILD-AA in 12 patients in the survival group (50%) and seven patients in the dead group (58.3%). Changes in KL-6 and SP-D values between these two time points (2 weeks after the onset of ILD-AA and the onset of ILD-AA) are shown in Fig. 4. Changes in SP-D levels in the 12 patients in the survival group were significantly decreased when compare to those from the seven patients in the dead group (−126 ± 67.91 ng/mL in the survival group versus 284 ± 114.30 ng/mL in the dead group, *p = 0.033*). In contrast, the change in KL-6 was not significantly different between two groups (137 ± 147.62 U/mL in the survival group versus 314 ± 208.77 U/mL in the dead group, *p = 0.752*).

### ΔSP-D cut off level and survival time

**Fig. 5:**
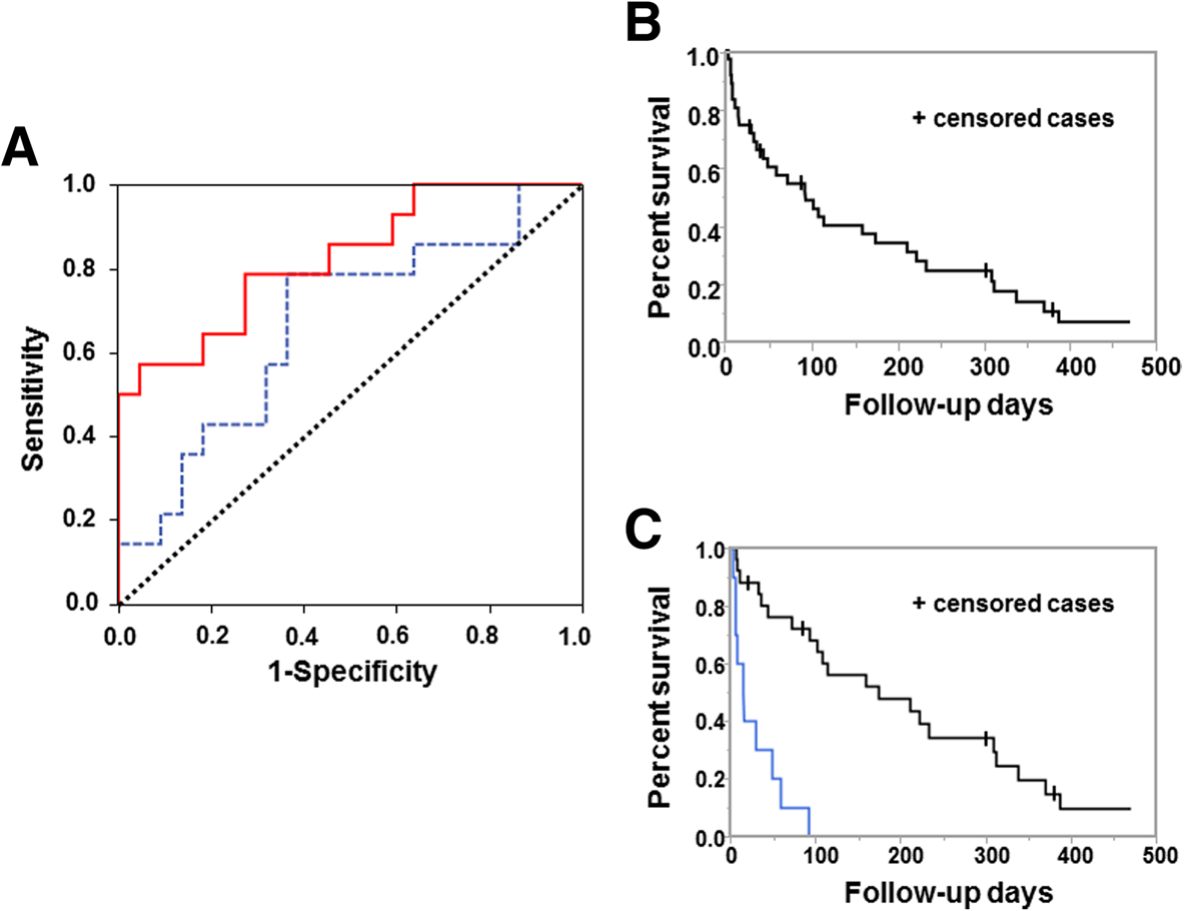
Receiver operating characteristic curve analysis of Δ serum markers and overall survival of patients with interstitial lung disease induced by anticancer agents. **a** Receiver operating characteristic (ROC) curve analyses to determine the optimal cut off values of ΔKL-6 (*blue line*) and ΔSP-D (*red line*) for predicting survival in patients with ILD-AA. Sensitivity, or true positive rate, is plotted on the y-axis, and false positive rate, or 1-specificity, on the x-axis. The area under the curve (AUC) is equivalent to the numerator of the Mann-Whitney U statistic comparing the marker distributions between the survival and the death group after diagnosis of ILD-AA (AUC, 0.825; 95% Confidence interval (CI), 0.68–0.97; *p = 0.001*). The optimal cut off value of ΔSP-D was 398 ng/mL, with a sensitivity, specificity, and likelihood ratio of 42.86%, 95.55%, and 9.52, respectively. The AUC is equivalent to the numerator of the Mann-Whitney U statistic comparing the marker distributions between the survival and the death group after the onset of ILD-AA (AUC, 0.669; 95% CI, 0.48–0.85; *p =* 0.092). The optimal cut off value for ΔKL-6 was 219 U/mL, with a sensitivity, specificity and likelihood ratio of 78.57%, 63.64%, and 2.16, respectively. **b** Survival time in total patients. Median survival time (MST) was 93 days in all patients diagnosed with ILD-AA (95% CI 36–174). **c** Difference of survival time between high and low ΔSP-D. Survival time for patients with low ΔSP-D was significantly longer than that for patients with high ΔSP-D (MST, 159 days; 95% CI﻿, 72–328 in low ΔSP-D [blue line] versus MST, 30 days; 95% CI, 3–33 in high ΔSP-D [black line], HR: 26.02, *p = 0.001*, by log-rank test)

To obtain optimal cut off values for ΔKL-6 and ΔSP-D in serum for prognostic assessments in patients with ILD-AA, a ROC curve analysis was performed using the highest ΔKL-6 and ΔSP-D values (Fig. 5a). To predict the risk of mortality within 6 weeks of the onset of ILD-AA, the optimal cut off value for ΔSP-D was 398 ng/mL. The cut off value of ΔKL-6 was nonsignificant because of a low likelihood ratio and area under the curve. Eight of the nine (88.9%) patients with high ΔSP-D (≥ 398 ng/mL) died, and six of the 27 (22.2%) patients with low ΔSP-D (< 398 ng/mL) died (*p* = 0.0003).

Median survival time (MST) was 93 days in all of the patients diagnosed with ILD-AA (95% CI, 36–174; Fig. 5b). Survival time for the patients with low ΔSP-D was significantly longer than that for the patients with high ΔSP-D (MST, 159 days; 95% CI, 72–328 in low ΔSP-D versus MST, 30 days, 95% CI, 3–33 in high ΔSP-D, HR: 26.02, *p* = 0.001, Fig. 5c).

### The risk factor for ILD-AA-related death

The results of univariate and multivariate analyses of the risk factors for death associated with ILD-AA are shown in Tables 3 and 4. In univariate analysis, high ΔSP-D and smoking history were significantly associated with ILD-AA-related death (high ΔSP-D: odds ratio [OR], 7.00; 95% CI, 2.19–72.26; *p* = 0.001 and smoking history: OR, 2.48; 95%CI, 1.26–4.86; *p* = 0.042, respectively). Multivariate analysis performed using six variables (age, smoking history, performance status, the presence of emphysema, the presence of interstitial shadow, and high ΔSP-D) showed that only high ΔSP-D was a significant independent risk factor for ILD-AA-related death (OR, 25.56; 95% CI, 2.29–285.46; *p* = 0.008).

**Table 3:** Univariate analysis of risk factors associated with death related to interstitial lung disease induced by anticancer agents

	Overall	Survival	Death	Odds ratio	95% CI	*p*
Overall	36	22	14			
Age				2.45	0.64–9.37	0.310
≤ 70	19	12	7			
≥ 71	17	10	7			
Sex				0.74	0.16–3.39	0.693
Female	9	5	4			
Male	27	17	10			
Performance status				1.22	0.44–3.43	0.693
0–1	27	16	11			
2–4	9	6	3			
Smoking history				2.48	1.26–4.86	0.042
No	5	1	4			
Yes	31	21	10			
With emphysema				1.50	0.67–3.39	0.334
No	13	6	7			
Yes	23	16	7			
With pre-existing interstitial shadow				1.67	0.73–3.81	0.221
No	15	8	7			
Yes	21	14	7			
ΔSP-D				7.00	2.19–72.26	0.001
< 398	27	21	6			
≥ 398	9	1	8			

*Abbreviations: CI* confidence interval, *SP-D* surfactant protein-D

**Table 4:** Multivariate analysis of risk factors associated with death related to interstitial lung disease induced by anticancer agents

	Odds ratio	95% CI	*P*
Variable			
Age (≤ 70 vs ≥ 71)	2.39	0.35–16.31	0.375
Smoking history (no vs yes)	7.69	0.38–20.41	0.174
Performance status (0–1 vs 2–4)	1.06	0.12–7.35	0.957
With emphysema (no vs yes)	1.66	0.18–15.15	0.653
With pre-existing interstitial shadow (no vs yes)	1.19	0.15–9.26	0.639
ΔSP-D (< 398 vs ≥ 398)	25.56	2.29–285.46	0.008

*Abbreviations: CI* confidence interval, *SP-D* surfactant protein-D

## Discussion

To our knowledge, this is the first study to document that an increase in serum SP-D is a significant biomarker for ILD-AA in patients with advanced lung cancer and SP-D elevation was significantly associated with ILD-AA-related death. In the patients diagnosed with ILD-AA, the incidence of death in those with high ΔSP-D (≥ 398 ng/mL) was significantly higher when compared to patients with low ΔSP-D (< 398 ng/mL). In addition, ΔSP-D was the only risk factor for death related to ILD-AA. Although we have previously used KL-6 for analysis ILD-AA diagnosis and follow-up, we consider SP-D to be superior to KL-6, and that it should be used for evaluation of prognosis and follow-up of ILD-AA.

We generally diagnose ILD-AA by several examinations, including HRCT findings, pulmonary function test, BAL fluid analysis, and pathological findings [11]. It is important to distinguish between ILD-AA and other types of ILD, including lymphangitis induced by cancer progression. In our research, we performed HRCT for all patients suspected of ILD-AA. However, we could not perform pulmonary function test, BAL, and transbronchial lung biopsy at the time of diagnosis with ILD-AA because of poor respiratory condition in other patients. Therefore, we could not evaluate ILD-AA development by these examinations and association between ΔSP-D and examination results, including pulmonary function test, BAL fluid, and pathological findings by transbronchial lung biopsy in this research.

To distinguish between ILD-AA and carcinomatous lymphangitis, we evaluated HRCT findings and any tumor markers related to lung cancer. If the patients developed lymphangitis, we could observe characteristic findings of lymphangitis, such as the thickening of bronchovascular bundle, interlobular septa, and centrilobular micro nodules. However, there was no evidence of lymphangitis in any patient diagnosed with ILD-AA. Next, we distinguished other causes of ILD, such as any infections, heart, or renal failure, and the fact that there were no newly administrated drugs besides anti-cancer agents. Therefore, we diagnosed these cases as ILD-AA based on HRCT findings, serum markers, and clinical course.

When compared with SP-B and SP-C, SP-D and SP-A have been reported to be crucial serum biomarkers for prognosis and disease activity in ILD [12]. Increased serum SP-A and SP-D are significantly associated with acute exacerbation of IIPs, including idiopathic pulmonary fibrosis (IPF) [12]. Initial serum SP-D levels in patients who die are significantly higher than those in patients who survive; this is in agreement with the results of our study. KL-6 has the highest specificity and sensitivity for ILDs [13, 14]. Serum KL-6 levels in patients with HP are higher than those in patients with IPF, CTD-IP, and sarcoidosis [15]. No previous reports have compared KL-6 and SP-D for assessing the prognosis and progression of D-ILD in patients with advanced lung cancer.

Serum KL-6 levels are rarely elevated at the time of diagnosis in patients with adenocarcinoma [16]. In this research, serum KL-6 levels elevated from baseline in half of the patients before the diagnosis of ILD-AA because KL-6 was serum fibrotic marker and tumor marker. Moreover, of the 14 patients diagnosed with IPF, serum SP-D levels in 5 patients and serum KL-6 levels in 12 patients elevated before the diagnosis of ILD-AA. Although we confirmed stable disease and partial response in Response Evaluation Criteria in Solid Tumors (RECIST) at the time of diagnosis with ILD-AA in all patients, it was difficult to evaluate the association between development of ILD-AA and serum markers levels at the time of diagnosis with ILD-AA only. Accordingly, we evaluated the serum markers at the time of diagnosis with ILD-AA and before the diagnosis of ILD-AA and analyzed serum markers changing between two points (ΔSP-D and ΔKL-6). We verified that ΔSP-D was the most suitable for the evaluation of development of ILD-AA compared with any serum markers in any other time points. Therefore, we could assess changes in serum markers induced by ILD-AA except for cancer and fibrosis progression and did not define the time point of ΔKL-6 and ΔSP-D as the time of diagnosis with advanced lung cancer, but as the time after initiation of any anticancer agents.

Our research indicates that ΔSP-D is the most useful marker in patients with advanced lung cancer and ILD-AA. Ishikawa et al. investigated D-ILD in patients with chronic hepatitis C treated with pegylated interferon. Whereas KL-6 levels tended to increase after development of D-ILD on HRCT, SP-D levels significantly increased at the development of D-ILD [17]. Ishikawa et al. suggested SP-D is more useful than KL-6 in evaluating prognosis in patients with pegylated interferon-induced ILD. Although the primary disease and treatment differed between the current study and that of Ishikawa et al. [17], theirs supports our results in showing that SP-D is the most useful marker in D-ILD.

Few reports describe other serum biomarkers for D-ILD, except for KL-6, SP-A, and SP-D. Serum KL-6 to serum sialyl lewis X-I antigen ratio (K/S ratio) has been reported to be a useful predictive marker for D-ILD in patients with lung cancer and ILD [18]. In this report, high K/S ratio (> 20), which was determined before the first-line chemotherapy, tended to increase the risk of D-ILD. Moreover, serum ADAM8 (a disintegrin and a metalloproteinase 8) concentrations were significantly elevated in patients with suspected drug-induced eosinophilic pneumonia induced by suspect drugs [19]. However, since measurement of ADAM8 is uncommon, and determining K/S ratio requires the measurement of two types of markers, ΔSP-D may be a more useful marker because clinicians need to measure only SP-D to calculate ΔSP-D.

Finally, ΔSP-D may be associated with prognosis of ILD-AA and DAD on HRCT. Serum SP-A and SP-D tend to be higher in patients who die of acute respiratory distress syndrome [20]. Thus, lung injury may occur in Type II pneumocytes in the alveolar epithelium and result in the release of SP-D into serum. The mortality rate in patients exhibiting DAD on HRCT is significantly higher than that in patients with other patterns who develop gefitinib-related ILD [21]. These reports also support our results, suggesting that elevation of serum SP-D is associated with a poorer prognosis and DAD development. However, to our knowledge, the association between other types of ILD on HRCT and serum SP-D changing is unknown [22].

This study had several limitations. First, this was a small retrospective study. The incidence of ILD-AA has been reported at approximately 3% in Japanese people and approximately 1% in all other nations. Thus, we calculated a quite large 95% CI because of a small number of patients. Second, the patients’ backgrounds were heterogeneous and various types of anticancer agents induced ILD-AA. Hence, this study included several different types of histology. However, because few patients were treated with each regimen, we were unable to calculate and evaluate differences in serum SP-D level and ΔSP-D between regimens. Thirdly, the diagnosis of ILD-AA was based on HRCT and laboratory findings, but not on histological findings. However, it is difficult to diagnose ILD pathologically through transbronchial or surgical biopsy because ILD often results in worsening of the respiratory condition.

## Conclusion

This is the first study to evaluate high ΔSP-D (≥ 398 ng/mL) in patients with ILD-AA and to determine the risk factors for ILD-AA in advanced lung cancer patients. The findings suggested that ΔSP-D was the only significant risk factor for mortality in patients with ILD-AA, and that associated with HRCT findings. ΔSP-D might be a predictive prognostic biomarker of ILD-AA.

## Abbreviations

ADAM8: A disintegrin and a metalloproteinase 8
AMR: Amrubicin
BEV: Bevacizumab
CBDCA: Carboplatin
CDDP: Cisplatin
CI: Confidence interval
CIP: Chronic interstitial pneumonia
CTD-IP: Connective tissue diseases associated with interstitial pneumonia
DAD: Diffuse alveolar damage
D-ILD: Drug-induced interstitial lung disease
DOC: Docetaxel
EGFR: Epidermal growth factor receptor
GGO: Ground glass opacity
HP: Hypersensitivity pneumonia
HR: Hazard ratio
HRCT: High resolution computed tomography
IIPs: Idiopathic interstitial pneumonias
ILD: Interstitial lung disease
ILD-AA: Interstitial lung diseases induced by anticancer agents
IPF: Idiopathic pulmonary fibrosis
K/S ratio: KL-6 to serum sialyl lewis X-I antigen ratio
KL-6: Krebs von den Lungen-6
MST: Median survival time
nab-PAC: Albumin-binding paclitaxel
NGT: Nogitecan
NS: No significant difference
NSCLC: Non-small cell lung cancer
OP/EP: Organized pneumonia/eosinophilic pneumonia
OR: Odds ratio
PAC: Paclitaxel
PEM: Pemetrexed
RECIST: Response Evaluation Criteria in Solid Tumors
ROC: Receiver operating characteristic
SCLC: Small cell lung cancer
SP-A: Surfactant protein-A
SP-D: Surfactant protein-D
VP-16: Etoposide
